# Sulfasalazine Treatment Suppresses the Formation of HLA-B27 Heavy Chain Homodimer in Patients with Ankylosing Spondylitis

**DOI:** 10.3390/ijms17010046

**Published:** 2015-12-29

**Authors:** Hui-Chun Yu, Ming-Chi Lu, Kuang-Yung Huang, Hsien-lu Huang, Su-Qin Liu, Hsien-Bin Huang, Ning-Sheng Lai

**Affiliations:** 1 Department of Medical Research, Dalin Tzu Chi Hospital, Buddhist Tzu Chi Medical Foundation, Chia-Yi 62247, Taiwan; df928039@tzuchi.com.tw; 2 Division of Allergy, Immunology and Rheumatology, Department of Medicine, Dalin Tzu Chi Hospital, Buddhist Tzu Chi Medical Foundation, Chia-Yi 62247, Taiwan; dm252940@tzuchi.com.tw (M.-C.L.); hky0919@yahoo.com.tw (K.-Y.H.); df897226@tzuchi.com.tw (S.-Q.L.); 3 School of Medicine, Tzu Chi University, Hualien 97004, Taiwan; 4 Department of Nutrition and Health Science, Fooyin University, Kaohsiung 83102, Taiwan; estrus@mail2000.com.tw; 5 Department of Life Science and Institute of Molecular Biology, National Chung Cheng University, Chia-Yi 62102, Taiwan

**Keywords:** ankylosing spondylitis, HLA-B27 homodimer, sulfasalazine, mean grade of sarcoiliitis and lumbar spine BASRI scores

## Abstract

Human leukocytic antigen-B27 heavy chain (HLA-B27 HC) has the tendency to fold slowly, in turn gradually forming a homodimer, (B27-HC)_2_ via a disulfide linkage to activate killer cells and T-helper 17 cells and inducing endoplasmic reticulum (ER) stress to trigger the IL-23/IL-17 axis for pro-inflammatory reactions. All these consequences lead to the pathogenesis of ankylosing spondylitis (AS). Sulfasalazine (SSA) is a common medication used for treatment of patients with AS. However, the effects of SSA treatment on (B27-HC)_2_ formation and on suppression of IL-23/IL-17 axis of AS patients remain to be determined. In the current study, we examine the (B27-HC)_2_ of peripheral blood mononuclear cells (PBMC), the mean grade of sarcoiliitis and lumbar spine Bath Ankylosing Spondylitis Radiology Index (BASRI) scores of 23 AS patients. The results indicated that AS patients without (B27-HC)_2_ on PBMC showed the lower levels of mean grade of sarcoiliitis and the lumbar spine BASRI scores. In addition, after treatment with SSA for four months, the levels of (B27-HC)_2_ on PBMCs were significantly reduced. Cytokines mRNA levels, including TNFα, IL-17A, IL-17F and IFNγ, were also significantly down-regulated in PBMCs. However, SSA treatment did not affect the levels of IL-23 and IL-23R mRNAs.

## 1. Introduction

Ankylosing spondylitis (AS) is an inflammatory disease that is characterized by inflammatory back pain and asymmetric peripheral oligoarthritis [1,2,3,4]. The development of AS is strongly associated with the expression of human leukocytic antigen-B27 (HLA-B27) [5,6]. HLA-B27 is one of the major histocompatibility complex (MHC) class I molecules. It consists of a heavy chain (α chain) and β_2_ microglobulin (β_2_m) and is assembled with an antigenic peptide in the endoplasmic reticulum (ER). The antigenic peptide-bound MHC class I complex is allowed to leave the ER and be transported to the cell surface for antigen presentation to CD8^+^ T cells.

The HLA-B27 heavy chain (HLA-B27 HC) has a propensity to misfold slowly in the ER before it is assembled with β_2_m and a peptide and forms a disulfide-linked heavy-chain homodimer, (B27-HC)_2_, that can be displayed on cell surfaces [7,8,9,10,11,12,13]. The abnormal (B27-HC)_2_ can be recognized by the killer-cell Ig-like receptor (KIR3DL2) on natural killer cells [14,15]. In addition, KIR3DL2 is also present on the membranes of T-helper 17 cells (Th17). Therefore, (B27-HC)_2_ is capable of stimulating IL-17 production by Th17 cells. These events possibly provide a linkage between the presence of (B27-HC)_2_ and the pathogenesis of AS [14,15,16].

B27-HC misfolding can induce ER stress and increase signaling of the IL-23/IL-17 axis. This finding is based on a recent study that used transgenic rats that over-expressed HLA-B27/human β_2_m [17]. HLA-B27 HC misfolding induces ER stress and results in activation of the unfolded protein response (UPR), which in turn stimulates NF-κB activation to increase the expression of pro-inflammatory cytokines such as TNFα, IL-1, IL-6, and IL-23 [18,19,20,21,22]. IL-23 is a key stimulus for the survival and activation of Th17 cells. Activated Th17 cells secrete IL-17 that stimulates IL-17-responsive cells to trigger the pro-inflammatory reaction. HLA-B27 misfolding is closely linked to Th17 cells through the activation of the UPR, and also provides a plausible mechanism for the pathogenesis of AS.

Sulfasalazine (SSA) is a non-steroidal anti-inflammatory agent that inhibits IκB kinases α and β [23,24] and is used to treat patients with peripheral arthritis. The inhibition of IκB kinase (IKK) activities by SSA results in maintenance of NF-κB in an inactive cytoplasmic complex with IκB, thus blocking the translocation of NF-κB from the cytosol to the nucleus. In this study, we analyzed the prevalence of (B27-HC)_2_ on peripheral blood mononuclear cells (PBMCs), the mean grade of sarcoiliitis and the lumbar spine Bath Ankylosing Spondylitis Radiology Index (BASRI) scores of 23 AS patients. We also examined the effects of SSA treatments on the production of (B27-HC)_2_ and mRNA levels of pro-inflammatory cytokines. Our results demonstrated that SSA treatment caused down-regulation of pro-inflammatory cytokine mRNA expression and reduced the production of (B27-HC)_2_ on PBMCs. However, SSA treatment did not affect the expression of IL-23 and IL-23 receptor (IL-23R) mRNA.

## 2. Results

### 2.1. Most of Ankylosing Spondylitis (AS) Patients Display (B27-Heavy Chain (HC))_2_ on Their Peripheral Blood Mononuclear Cells (PBMCs), and the Mean Grades of Sacroiliitis and the Lumbar Spine Bath Ankylosing Spondylitis Radiology Index (BASRI) Scores Are Lower in AS Patients without (B27-HC)_2_ on Their PBMCs

**Figure 1:**
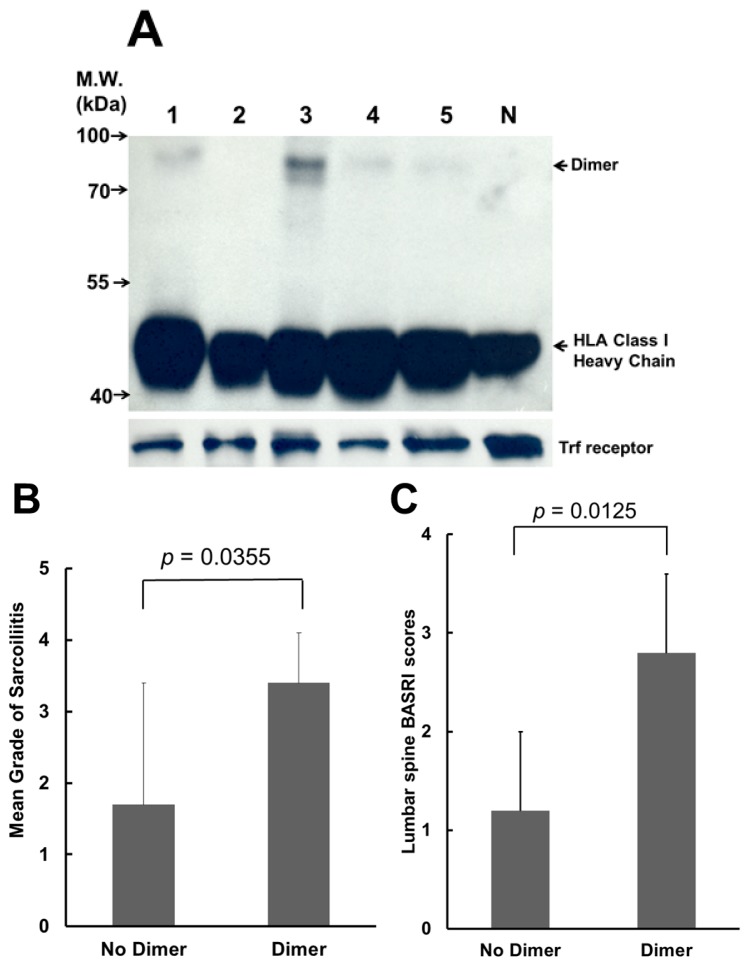
Analysis of (B27-heavy chain (HC))_2_ of ankylosing spondylitis (AS) patients. (**A**) Most of the AS patients have (B27-HC)_2_ on their membrane of peripheral blood mononuclear cells (PBMCs). Lanes **1**–**5**: membrane proteins extracted from PBMCs of AS patients. Lane **N**: membrane proteins extracted from PBMCs of a healthy control without expression of human leukocytic antigen-B27 (HLA-B27). Membrane proteins (50 µg) from individual patients were loaded in each lane, separated by non-reducing sodium dodecyl sulfate polyacrylamide gel electrophoresis (SDS-PAGE) (12%), and analyzed by western blot using BH2. Internal control: transferrin receptor (Trf receptor); M.W.: molecular weight; (**B**) Analysis of the mean grade of sacroiliitis from AS patients with or without (B27-HC)_2_ on their membranes of PBMCs (*p* < 0.05 by Mann–Whitney *U* test); and (**C**) Analysis of the grade of lumbar spine Bath Ankylosing Spondylitis Radiology Index (BASRI) scores from AS patients with or without (B27-HC)_2_ on their membranes of PBMCs (*p* < 0.05 by Mann–Whitney *U* test).

BH2 is a monoclonal antibody that can recognize the misfolded (B27-HC)_2_ [25,26]. The recognition site of BH2 is located within the α_2_ domain of B27-HC [26]. We analyzed (B27-HC)_2_ extracted from the membranes of PBMCs by western blot using BH2 monoclonal antibody. Figure 1A shows the analysis of (B27-HC)_2_ on PBMCs obtained from five AS patients. Of the 23 AS patients, 18 (78%) displayed (B27-HC)_2_ on their PBMCs. AS patients lacking the (B27-HC)_2_ on their PBMCs exhibited a lower mean grade of sacroliitis (Figure 1B) and lower lumbar spine BASRI scores (Figure 1C).

### 2.2. Sulfasalazine (SSA) Treatment Suppressed the Production of (B27-HC)_2_

**Figure 2:**
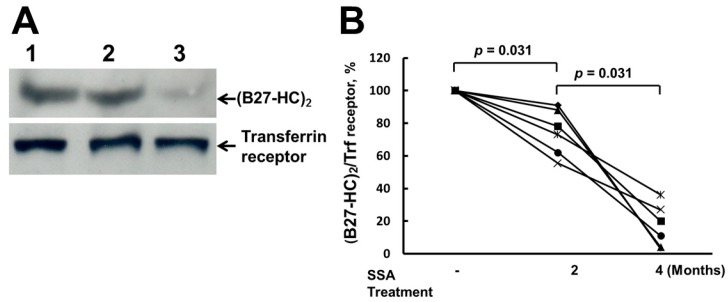
SSA suppressed the formation of (B27-HC)_2_. (**A**) Western blot analysis of (B27-HC)_2_ extracted from PBMCs from a representative AS patients before and after SSA treatment. Lane **1**: 50 µg membrane proteins before SSA treatment; Lane **2**: 50 µg membrane proteins after SSA treatment for two months; Lane **3**: 50 µg membrane proteins after SSA treatment for four months. Internal control: transferrin receptor; (**B**) SSA treatment reduced the production of (B27-HC)_2_ (*p* < 0.05 by Wilcoxon signed rank test). Based on the results of Figure 2A, the level of (B27-HC)_2_ on PBMCs from a single AS patient before SSA treatment was set at 100%. For each AS patient, the percent of (B27-HC)_2_ after SSA treatment was compared with that before SSA treatment. Data were obtained from six AS patients.

Levels of (B27-HC)_2_ were examined after AS patients were treated with SSA. The amount of (B27-HC)_2_ on PBMCs of AS patients was reduced after two months of SSA treatments, and was further significantly reduced after 4 months of treatment (Figure 2A). Six AS patients who displayed (B27-HC)_2_ on PBMCs were monitored over time after SSA treatments, and their levels of (B27-HC)_2_ on PBMCs were reduced after two and four months of treatment with SSA, respectively (Figure 2B).

### 2.3. SSA Repressed the Expression of TNFα, IL-17A, IL-17F, and IFNγ mRNA, but Did Not Affect the Levels of mRNA of IL-23 and IL-23R

**Figure 3:**
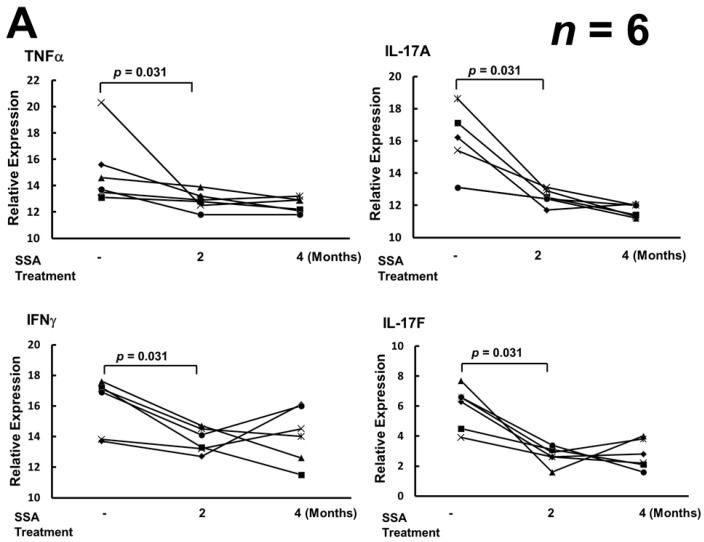

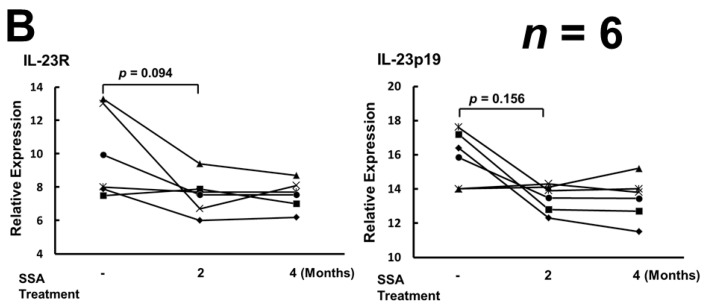
The effects of SSA treatment on mRNA levels of pro-inflammatory cytokines and IL-23 receptor (IL-23R). (**A**) SSA treatment suppressed the expression of TNFα, IL-17A, IL-17F, and IFNγ mRNA (*p* < 0.05 by Wilcoxon signed rank test); (**B**) SSA treatment had no effect on the levels of IL-23 and IL-23R mRNA. Real-time RT-PCR values for each inflammatory cytokine were normalized to those of 18S rRNA (*p* > 0.05 by Wilcoxon signed rank test). Data were obtained from six AS patients before SSA medication, after SSA medication for two months, and after SSA medication for four months, respectively.

Real-time polymerase chain reaction (RT-PCR) was used to determine whether SSA treatments down-regulated the transcription of the pro-inflammatory cytokines of AS patients via inactivation of NF-κB. SSA treatments reducing the levels of TNFα, IL-17A, and IL-17F mRNA in PBMCs from AS patients (Figure 3A). Down-regulation of IL-17A/F transcripts was observed after treatment for two months. In addition, SSA treatments also suppressed the levels of IFNγ mRNA (Figure 3A). However, SSA treatments did not affect the levels of IL-23 and IL-23R mRNA in PBMCs from AS patients (Figure 3B).

## 3. Discussion

The ER is the organelle responsible for protein folding, glycosylation, trafficking, and targeting. Accumulation of newly-synthesized unfolded/misfolded proteins can overload the protein cargo capacity of the ER and increase ER stress to activate the UPR [17,18,19]. In the transgenic rat model, overexpression of HLA-B27 could promote ER stress, activate the UPR and stimulate the IL23/IL17 axis to induce the pro-inflammatory reaction [17]. However, UPR induced by overexpressed HLA-B27 was not observed in macrophages obtained from AS patients [27]. SSA blocks the transcription of NF-κB-regulated pro-inflammatory cytokines [19,23,24]. In our study, mRNA levels of the pro-inflammatory cytokines IFNγ and TNFα in PBMCs were down-regulated following SSA treatment (Figure 3A). Lower levels of IFNγ and TNFα could reduce the transcription of HLA-B27 HC and decrease the formation of (B27-HC)_2_ [1]. Th17 cells in AS patients can be activated by (B27-HC)_2_, which activates the KIR3DL2 receptor. In our results, IL-17A and IL-17F mRNA expression in activated Th17 cells was reduced in AS patients following SSA treatment (Figure 3A). However, SSA treatment did not affect the levels of IL-23 and IL-23R mRNA in PBMCs of AS patients (Figure 3B). Down-regulation of IL-17A and IL-17F mRNA expression may arise from the decreased stimulation of Th17 cells by (B27-HC)_2_ molecules, the number of which displayed on the cell membrane of PBMCs was decreased after SSA treatment (Figure 2A,B), or from the blockage of NF-κB signaling [23,24].

More than 30% of AS patients develop typically severe spinal destruction over time [28,29]. Radiological changes to the lumbar spine and sacroiliac point are important markers for AS diagnosis. Our results indicated that AS patients without (B27-HC)_2_ on their PBMCs showed a lower mean grade of sacroiliitis and lower lumbar spine BASRI scores, suggesting that (B27-HC)_2_ might correlate with the development of severe spine restriction. The effect of (B27-HC)_2_ on spinal damage was unknown and needed to be characterized. Although SSA treatment can reduce the levels of (B27-HC)_2_, the spinal damage cannot be reversed after treatment.

BH2 used in this study is a monoclonal antibody that can recognize most HLA-B and HLA-C allelic heavy chains [26]. Our previous study also suggested that BH2 may bind to HLA allelic heavy chains with different affinity. Therefore, the band representing the monomeric form of MHC class I heavy chain in Figure 1A that displays a different density could result from the different affinity with BH2. However, the homodimeric form of HLA-B27 heavy chain is homogeneous. The band density of homodimeric HLA-B27 heavy chain in Figure 1A can reflect the levels of the dimeric protein.

Two types of MHC class I heavy chain that can form the homodimer via the disulfide bond located at the α1 domain have been identified [30]. They are HLA-B27 and HLA-G. However, the non-classical MHC class I molecule, HLA-G, is mainly expressed in the fetal placental trophoblast cells [30]. Recently, a third type of dimer MHCI molecule, termed as MHC class I redox-induced dimers, has been identified, primarily localized on exosomes or on apoptotic cells [30,31]. The disulfide linkage in these exosomal dimers is formed in the cytoplasmic tail domain region. Therefore, the mixed-allele dimers could form under these conditions. KIR3DL2 recognition by MHC class I redox-induced dimers needs to be characterized. However, in our healthy control, no mix-allele dimers were observed (Figure 1A). In addition, only a homodimeric form of the HLA-B27 heavy chain has been identified in PBMCs isolated from the AS patient [12]. No heterodimers, formed by the HLA-B27 heavy chain linked with other HLA heavy chain, have been observed.

## 4. Experimental Section

### 4.1. Materials

Sodium dodecyl sulfate (SDS), acrylamide, 2-mercaptoethanol, iodoacetamide, *N*,*N*,*N*’,*N*’-tetramethyl-ethylenediamine, ammonium persulfate, Dithiothreitol (DTT), glycine and Tris Base were purchased from Sigma-Aldrich (St. Louis, MO, USA).

### 4.2. Patients

Twenty-three patients with AS who expressed the HLA-B27 protein were enrolled in this study. Patients were defined according to the modified New York criteria [32]. Enrollment was carried out from June, 2012 to June, 2013 in Buddhist Dalin Tzu-Chi General Hospital, Chia-Yi, Taiwan. All participators signed informed consent forms approved by the Institutional Review Board and Ethics Committee (IRBEC) of Buddhist Dalin Tzu-Chi General Hospital. The method for isolation of human PMBCs from AS patients has been reviewed and approved by IRBEC. The IRBEC approval number for the certificate issued in June 2012 is B09801021-1. A written informed consent document has been obtained from each participator. AS patients were treated with sulfasalazine (250 mg/day–2 g/day). Blood samples from six (B27-HC)_2_-positive AS patients were collected after two and four months of treatment with SSA for analysis of (B27-HC)_2_ and the expression of pro-inflammatory cytokine mRNAs.

### 4.3. Western Blot Analysis

Human PBMCs were prepared as previously described [33]. Membrane proteins of PBMCs from AS patients and healthy controls were extracted using a ProteoExtract Native Membrane Protein Extraction Kit (Calbiochem, Darmstadt, Germany). Freshly prepared iodoacetamide (10 mM) was applied in all steps to block the formation of disulfide bridges during membrane protein extraction. Supernatant containing the extracted membrane proteins was collected and 50 µg of extracted membrane proteins were resolved by 12% non-reducing SDS-PAGE, analyzed by western blotting and probed for (B27-HC)_2_ by BH2 monoclonal antibody [25,26]. The cognate molecules were visualized through a chemiluminescence reaction (Amersham Biosciences, Piscataway, NJ, USA).

### 4.4. Analysis of Grade of Sacroiliitis and BASRI Scores

The grade of sacroiliitis was identified according to the New York criteria [34] and the lumbar spine involvement was graded using the Bath Ankylosing Spondylitis Radiology Index (BASRI) scores [35] in AS patients.

### 4.5. Real-Time PCR

Total RNA was isolated from purified PBMCs of AS patients with a QIAamp RNA Blood Mini kit (QIAGEN, GmbH, Germany). IFNγ, TNFα, IL-17A, IL-17F, IL-23 19P or IL-23R mRNA was amplified by real-time PCR using a One Step SYBR Ex Taq qRT-PCR kit (TaKaRa, Shiga, Japan). The suitable primers were synthesized as described [36,37,38]. The resulting products were analyzed on an ABI 7500 Real Time PCR System (Applied Biosystems, Foster City, CA, USA).

### 4.6. Statistical Analysis

Statistical significance for the difference of sacroiliitis and BASRI scores from AS patients with and without (B27-HC)_2_ on their membranes of PBMCs was analyzed by the Mann–Whitney *U* test. The Wilcoxon signed rank test was used to compare the variation of (B27-HC)_2_ and cytokine mRNAs after SSA treatment. *p*-values < 0.05 were considered statistically significant.

## 5. Conclusions

We have demonstrated that most of the AS patients display (B27-HC)_2_ on their PBMCs and the mean grade of sacroiliitis and the grade of lumbar spine BASRI scores are higher when (B27-HC)_2_ is present on the PBMCs of AS patients. SSA treatment can suppress the levels of (B27-HC)_2_ and reduces the mRNAs of pro-inflammatory cytokines, such as IFNγ, TNFα, IL-17A and IL-17F, but is weak or has no effect on the levels of IL-23 and IL-23R mRNAs.
